# Lack of Secondary Transmission of Ebola Virus from Healthcare Worker to 238 Contacts, United Kingdom, December 2014

**DOI:** 10.3201/eid2312.171100

**Published:** 2017-12

**Authors:** Paul Crook, Alison Smith-Palmer, Helen Maguire, Noel McCarthy, Hilary Kirkbride, Bruce Court, Sanch Kanagarajah, Deborah Turbitt, Syed Ahmed, Paul Cosford, Isabel Oliver

**Affiliations:** Public Health England, London, UK (P. Crook, H. Maguire, N. McCarthy, H. Kirkbride, B. Court, S. Kanagarajah, D. Turbitt, P. Cosford, I. Oliver);; Health Protection Scotland, Glasgow, Scotland, UK (A. Smith-Palmer, S. Ahmed);; University College London, London (H. Maguire)

**Keywords:** Ebola virus disease, EVD, Ebola virus, viruses, airports, aircraft, contact tracing, healthcare worker, communicable diseases, hemorrhagic fever, travel, public health, secondary transmission, zoonoses, Sierra Leone, London, England, Scotland

## Abstract

In December 2014, Ebola virus disease (EVD) was diagnosed in a healthcare worker in the United Kingdom after the worker returned from an Ebola treatment center in Sierra Leone. The worker flew on 2 flights during the early stages of disease. Follow-up of 238 contacts showed no evidence of secondary transmission of Ebola virus.

More than 28,000 cases of Ebola virus disease (EVD) were reported during the epidemic in West Africa in 2014–2015 (*1*). International healthcare workers (HCWs) responded, and a number subsequently became infected with EVD, including those given a diagnosis after their return travel (*2*). There is international guidance for follow-up of aircraft contacts of case-patients with EVD (*3*–*5*).

We report contact tracing after EVD was diagnosed in an HCW in the United Kingdom who had worked in an Ebola treatment center in Sierra Leone. This worker had become ill while traveling back to the United Kingdom via Morocco.

## The Study

For this investigation, a multiagency incident control team and contact tracing teams were established with staff of Public Health England (PHE) and Health Protection Scotland. We undertook a risk assessment that included interviewing the case-patient about symptoms, travel, and contacts. We used preexisting guidance (*6*) to classify classified contacts according to their degree of exposure to the case-patient and to other persons with EVD on the basis of the degree of infectiousness and physical contact (Table). The degree of public health follow-up of contacts was formulated according to their exposure category (Table).

**Table:** Characteristics of 238 contacts of a healthcare worker with Ebola virus disease and summary of public health action, by exposure category, United Kingdom, December 2014*

Characteristic	No. contacts by exposure category			Total
	1; No direct contact	2; Direct contact with low risk for exposure	3; Direct contact with high risk for exposure	
Exposure setting				
Casablanca–LHR passengers	109	23	0	132
Casablanca–LHR crew	13	0	0	13
LHR PHE screeners	4	0	0	4
LHR Border Force	7	0	0	7
LHR–Glasgow passengers	62	8	0	70
LHR–Glasgow crew	7†	0	0	7
Healthcare workers	4‡	0	0	4
Glasgow community	0	1	0	1
Total§	185	24	29¶	238
Summary of public health action	Risk assessment questionnaire completed; provided telephone advice on symptoms of EVD; action to take if they had fever or symptoms consistent with EVD in the 21 d after their most recent exposure; advised that there was no reason to stop their day-to-day activities during the surveillance period provided they remained well; advised that their family and household contacts were not at risk for EVD if contact remained asymptomatic	As for category 1 plus provided written advice and a temperature monitoring kit, advised to take their temperature twice a day and to contact the local public health team if they had a fever or symptoms consistent with EVD in the 21 d after their most recent exposure; advised to delay any nonessential healthcare treatment until after the 21-d period; local public health teams assessed the contact after 21 d and reported their completion of public health follow-up	As for category 2 plus required to contact the local public health team daily to report their temperature and advised not to undertake healthcare activity; if contact was not made, the local public health team contacted them	NA

*In England a home visit was attempted if no telephone contact was possible. EVD, Ebola virus disease; LHR, London Heathrow Airport; NA, not applicable; PHE, Public Health England. †Includes 3 cabin staff and 4 land customer services staff. ‡Includes 2 doctors and 2 nurses who provided dedicated care to the patient while in the specialist infectious diseases unit. §Reflects overall number of persons categorized according to their highest category, including those categorized because of their healthcare work contact in Sierra Leone. Numbers in each category do not represent a sum of a column. ¶29 contacts who flew from Casablanca–LHR were classified as category 3 because of their healthcare-related activity in West Africa.

We asked airlines to provide passenger manifests; advanced passenger information (nationality, passport numbers, date of birth); and contact details. If contact details were missing or incorrect, we sought additional information from Her Majesty’s Passport Office, the UK Border Agency, PHE port entry screening and returning workers information, the National Health Service Patient Demographic Service, and online social networks. For contacts with UK addresses, we conducted a home visit if telephone contact was unsuccessful. For foreign national contacts, PHE also contacted the National Focal Point of the relevant country as per International Health Regulations. Public helplines were established to provide a contact point for those potentially affected. PHE provided public health advice to United Kingdom airline staff, and Moroccan authorities provided public health advice to Morocco airline staff. Moroccan authorities led contact tracing for the flight to Morocco, which is not reported in this article.

The case-patient was a UK national HCW (39-year-old woman) who had worked in an Ebola treatment center in Sierra Leone and had direct patient contact. On December 28, 2014, she flew from Sierra Leone to Glasgow on 3 flights and 2 aircraft of 2 airlines: from Freetown, Sierra Leone, to Casablanca, Morocco (3 h, 40 min); Casablanca to London, England (3 h, 30 min); and London to Glasgow, Scotland (1 h, 20 min).

During exit screening before boarding in Freetown and Casablanca, the case-patient was reported to have been apyrexial. Symptoms of fever and malaise began during the Casablanca to London flight. At London Heathrow Airport, several readings of temperatures <37.6°C were made by screening staff before onward travel. In Glasgow, after the case-patient took a taxi home, her symptoms worsened, and she telephoned local health services. The case-patient did not report vomiting, diarrhea, or bleeding before diagnosis and admission. Contacts had not been exposed to body fluids.

The case-patient was admitted to a hospital in Glasgow and given a diagnosis of EVD on December 29, 2014. The patient was subsequently transferred by military plane to a specialist infectious diseases hospital in London on December 30, 2014. She survived her illness.

Aircraft passengers in the same seat row and 2 rows in front of and behind the case-patient were classified as category 2 contacts. All other passengers and crew were classified as category 1 contacts. Border Force staff, health screeners, and HCWs were classified as category 1 contacts, and a community contact was classified as category 2 contact (Table).

A total of 238 persons, including 185 category 1 contacts and 24 category 2 contacts, were assessed as having sufficient exposure to the case-patient to warrant further public health follow-up (Table). Although no contacts were classified as being category 3 (direct contact with high exposure risk) because of exposure to the case-patient, 29 contacts were classified as category 3 because of other Ebola exposures, including healthcare work in Sierra Leone.

All 16 non–flight-related contacts were successfully followed up. Passengers on the Casablanca to London flight represented 17 nationalities, and 59 (45%) of 132 had non-UK passports. We successfully contacted all passengers on both flights, except for 3 category 1 passengers who had non-UK passports (199/202, 99%); a total of 162 (80%) passengers were contacted within 48 h of diagnosis for the case-patient.

We provided information for the 3 passengers we could not contact to their respective countries through their National Focal Point. Two persons in the United Kingdom required home visits. All flight crew were contacted. No additional cases of EVD were identified among contacts.

## Conclusions

We report no evidence of secondary transmission of Ebola virus to contacts of a case-patient who contracted EVD during the Ebola epidemic in West Africa in 2014–2015 and who was given a diagnosis in the United Kingdom. There have been few reports of symptomatic EVD case-patients traveling by commercial airline, and secondary transmission has not been documented (*7*–*10*). The case-patient we report was probably in the early stages of illness, and there was no evidence of high-risk exposure to body fluids on board the aircraft.

A precautionary approach was taken regarding classification of contacts on the aircraft. We used a wider definition for the closest contact category than that recommended by international guidance (1 seat in either direction of the case-patient) (*3*–*5*) but consistent with that used for the response to a case diagnosed in the United States (*10*). The decision to contact all persons on the affected aircraft was taken to inform and reassure contacts, not just to prevent transmission.

The limited information on passengers initially available hampered the ability to make contact promptly and resulted in extensive efforts to trace persons. Furthermore, flight manifests included passengers who had not flown. Issues with flight information have arisen before in the United Kingdom related to contact tracing (*11*). The need for airlines to collect personal rather than agency contact details should be stressed. To ensure a rapid response, public health agencies need to work with airlines and other international organizations to ensure access to a minimum dataset that would support rapid notification of contacts. In the United Kingdom, the National Health Service Patient Demographic Service was essential for obtaining contact details and identifying whether contacts were likely to be UK residents. In addition, the Ebola port entry screening and returning workers scheme provided rapid information on 37 contacts whose journey originated in Ebola-affected countries.

Temperature screening while the case-patient transferred through Heathrow Airport did not confirm fever, and the patient was allowed to continue the journey. In the presence of exit screening, airport entry screening might not detect imported EVD (*12*,*13*). However, one purpose of this screening has been to ensure that persons receive information to enable them to seek care quickly and in a manner that helps prevent transmission. Advice provided during airport screening and healthcare deployments aims to ensure that when symptoms develop, exposed persons contact health services early and by telephone, enabling responders to take appropriate measures to reduce transmission. In this instance, early contact with health services resulted in only a small number of contacts outside the flights. An evaluation of EVD port entry screening and a scheme for returning workers in the United Kingdom is under way.

This study provides support for the conclusion that there is low risk for transmission of Ebola virus on aircraft from EVD case-patients in the early stages of disease. Providing information and advice to contacts can be a helpful public health intervention in minimizing virus transmission.

## References

[1] World Health Organization. Ebola situation report, March 20, 2016 [cited 2017 Sep 14]. http://apps.who.int/ebola/current-situation/ebola-situation-report-30-march-2016

[2] Petti S, Protano C, Messano GA, Scully C. Ebola virus infection among western healthcare workers unable to recall the transmission route. Biomed Res Int. 2016;2016:8054709. Epub 2016 Nov 27. 10.1155/2016/8054709 PMC5149594 28018915

[3] Leitmeyer K. European risk assessment guidance for infectious diseases transmitted on aircraft—the RAGIDA project. Euro Surveill. 2011;16:19845. 21527131

[4] Gilsdorf A, Morgan D, Leitmeyer K. Guidance for contact tracing of cases of Lassa fever, Ebola or Marburg haemorrhagic fever on an airplane: results of a European expert consultation. BMC Public Health. 2012;12:1014. 10.1186/1471-2458-12-1014 23170851 PMC3533809

[5] World Health Organization. Travel and transport risk assessment: interim guidance for public health authorities and the transport sector, 2014 [cited 2017 Sep 14]. http://apps.who.int/iris/bitstream/10665/132168/1/WHO_EVD_Guidance_TravelTransportRisk_14.1_eng.pdf

[6] Advisory Committee on Dangerous Pathogens Public Health England. Management of hazard group 4 viral haemorrhagic fevers and similar human infectious diseases of high consequence, November 2015 [cited 2017 Sep 14]. http://www.nric.org.uk/node/54189

[7] Formenty P, Hatz C, Le Guenno B, Stoll A, Rogenmoser P, Widmer A. Human infection due to Ebola virus, subtype Côte d’Ivoire: clinical and biologic presentation. J Infect Dis. 1999;179(Suppl 1):S48–53. 10.1086/514285 9988164

[8] Shuaib F, Gunnala R, Musa EO, Mahoney FJ, Oguntimehin O, Nguku PM, et al.; Centers for Disease Control and Prevention (CDC). Ebola virus disease outbreak - Nigeria, July-September 2014. MMWR Morb Mortal Wkly Rep. 2014;63:867–72. 25275332 PMC4584877

[9] Fasina FO, Shittu A, Lazarus D, Tomori O, Simonsen L, Viboud C, et al. Transmission dynamics and control of Ebola virus disease outbreak in Nigeria, July to September 2014. Euro Surveill. 2014;19:20920. 10.2807/1560-7917.ES2014.19.40.20920 25323076

[10] Regan JJ, Jungerman R, Montiel SH, Newsome K, Objio T, Washburn F, et al.; Centers for Disease Control and Prevention (CDC). Public health response to commercial airline travel of a person with Ebola virus infection - United States, 2014. MMWR Morb Mortal Wkly Rep. 2015;64:63–6. 25632954 PMC4584560

[11] Parry-Ford F, Boddington N, Pebody R, Phin N, on behalf of the Incident Managemen C; Incident Management Team. Public health response to two incidents of confirmed MERS-CoV cases travelling on flights through London Heathrow Airport in 2014 – lessons learnt. Euro Surveill. 2015;20:21114. 10.2807/1560-7917.ES2015.20.18.21114 25990234

[12] Mabey D, Flasche S, Edmunds WJ. Airport screening for Ebola. BMJ. 2014;349(oct14 17):g6202. 10.1136/bmj.g6202 25316030

[13] Cosford P. Advantages of airport screening for Ebola. BMJ. 2014;349(nov04 4):g6585. 10.1136/bmj.g6585 25371224

