# Correction: Vol. 23, No. 12

**DOI:** 10.3201/eid2406.C22406

**Published:** 2018-06

**Keywords:** errata, erratum, correction

Patient data were inaccurate and definition of contact categories unclear in Lack of Secondary Transmission of Ebola Virus from Healthcare Worker to 238 Contacts, United Kingdom, December 2014 (P. Crook et al.). The article has been corrected online (https://wwwnc.cdc.gov/eid/article/23/12/17-1100_article).

